# A Personalized Genomics Approach of the Prostate Cancer

**DOI:** 10.3390/cells10071644

**Published:** 2021-06-30

**Authors:** Sanda Iacobas, Dumitru A. Iacobas

**Affiliations:** 1 Department of Pathology, New York Medical College, Valhalla, NY 10595, USA; sandaiacobas@gmail.com; 2 Personalized Genomics Laboratory, Center for Computational Systems Biology, Roy G Perry College of Engineering, Prairie View A&M University, Prairie View, TX 77446, USA

**Keywords:** apoptosis, ENTPD2, evading apoptosis, gene master regulators, genomic fabric, immortality, proliferation, P53 signaling, tumor heterogeneity

## Abstract

Decades of research identified genomic similarities among prostate cancer patients and proposed general solutions for diagnostic and treatments. However, each human is a dynamic unique with never repeatable transcriptomic topology and no gene therapy is good for everybody. Therefore, we propose the Genomic Fabric Paradigm (GFP) as a personalized alternative to the biomarkers approach. Here, GFP is applied to three (one primary—“A”, and two secondary—“B” & “C”) cancer nodules and the surrounding normal tissue (“N”) from a surgically removed prostate tumor. GFP proved for the first time that, in addition to the expression levels, cancer alters also the cellular control of the gene expression fluctuations and remodels their networking. Substantial differences among the profiled regions were found in the pathways of P53-signaling, apoptosis, prostate cancer, block of differentiation, evading apoptosis, immortality, insensitivity to anti-growth signals, proliferation, resistance to chemotherapy, and sustained angiogenesis. *ENTPD2, AP5M1 BAIAP2L1,* and *TOR1A* were identified as the master regulators of the “A”, “B”, “C”, and “N” regions, and potential consequences of *ENTPD2* manipulation were analyzed. The study shows that GFP can fully characterize the transcriptomic complexity of a heterogeneous prostate tumor and identify the most influential genes in each cancer nodule.

## 1. Introduction

According to the 29–31 March 2021 release of the Harmonized Cancer Datasets Genomic Data Commons Data Portal [1], 33,288 mutations in 20,237 genes were identified so far in 2355 cases of prostate cancer. The most frequently mutated genes in prostate cancer, *TP53* (tumor protein p53) and *TTN* (titin) are also among the most 10 frequently mutated genes in almost all other cancers. Although its long DNA (304.814 kb [2]) makes *TTN* more vulnerable to random alterations, *TP53*, with “only” 25.760 kb [3] is even more frequently mutated. Anyhow, together with the blamed mutation, millions of others occur in each individual, whose contributions to the cancer pathology are neglected without evidence that they are really negligible. 

Transcriptomic data from prostate tumors were compared to identify similarly regulated genes (e.g., [4,5]) whose restoration might provide therapeutic solutions. Again, unrepeatable combinations of hundreds of other genes are regulated among patients, and their contributions are unjustifiably neglected. 

Several commercially available cancer diagnostic assays compare either the base sequences or the expression levels of selected genes with corresponding “standard” mutation panels (e.g., [6,7,8,9,10]) or “standard” transcriptomic signatures (e.g., [11,12,13]). However, how universal is the “standard” derived from meta-analyses of gene sequences or/and expression levels in large populations of healthy and cancer individuals (e.g., [14,15,16,17,18,19])?

Many papers, including ours, proved that gene expression profiles depend on the genetic background [20], sex [21], age [22], hormones [23], disease and treatment [24,25], and environmental conditions [26,27,28,29]. Owing to the astronomic number of potential combinations, it is impossible to find two individuals with the same combination of regulatory factors and identical influences of these factors. Thus, the same cancer phenotype may be associated with distinct transcriptomes in different individuals as we reported for two cases of metastatic prostate cancers [30]. Although histologically similar, the unrepeatable combination of the “other” regulated/mutated genes makes the cancer of each individual unique. Therefore, whenever possible, one should refer the gene expression profile in cancer nodules to that of the surrounding cancer-free tissue of the same tumor (e.g., [31,32,33]). 

In recent years, highly cited papers documented that tumors have not only heterogeneous histology but also heterogeneous gene expression profiles [34,35,36] as proved also by us in a case of clear cell renal cell carcinoma [37]. If this is true within one tumor, what justifies comparing the average gene expression profiles in large groups of phenotypically similar but distinct cancer patients with healthy counterparts as reported by thousands of papers? 

Several specialized software text-mined the literature to select the genes that may have a role in prostate cancer. For instance, the Kyoto Encyclopedia of Genes and Genomes (KEGG, [38]) selected 97 genes involved in the prostate cancer (PRC) pathway [39] out of which we quantified 84.

## 2. Materials and Methods

### 2.1. Genomic Fabric Paradigm

Almost all cancer genomists limit their analyses to identifying what gene was up-/down-regulated in cancer with respect to normal tissue. Rather, we adopted the Genomic Fabric Paradigm (GFP) [40] that considers the transcriptome as a multi-dimensional mathematical object subjected to dynamic sets of transcripts’ abundances controls and expression correlations among the genes. Instead of the most frequently regulated biomarker genes in large populations of cancer patients, GFP looks for genes with commanding roles in the cancer of THIS person NOW. Being a personalized approach, GFP is not based on meta-analyses, and instead of universal gene targets, it identifies what should be done for the current patient. 

GFP makes full use of profiling tens of thousands of transcripts at a time in several biological replicates. It characterizes every gene by average expression level (AVE), Relative Expression Variability (REV) across biological replicas, and expression correlation (COR) with each other gene from the same region and with any gene from other regions. Comparing the AVE values in two regions determines what gene was significantly regulated. 

The separately profiled biological replicas are like instances of the same system subjected to slightly different environmental conditions. Thus, the expression variability among biological replicas provided an indirect estimate of how much the stability of transcripts’ abundances are controlled by the cellular homeostatic mechanisms [30]. Genes whose right expression is critical for the cell’s normal behavior are kept under stricter control and protected against environmental fluctuations, while genes empowering the cell adaptation to the environmental irregular changes are left to adjust. 

The simultaneous quantification of thousands of genes across biological replicas allows determining how much expression of one gene is coordinated with the expression of any other gene from the same or from another region. The expression correlation analysis is based on the “Principle of Transcriptomic Stoichiometry” [41], a generalization of Proust’s Law of Definite Proportions and Dalton’s Law of Multiple Proportions from chemistry [42], requiring the expression coordination of all genes whose encoded products are involved in a functional pathway. Expression correlation of genes does not stop at the cell boundary, the transcellular transcriptomic networks being essential for the integration and synchronization of multi-cellular structures as proved by us in the brain pan-glial transcriptomic continuity [43]. The use of these three independent groups of features increases the workable information provided by a high throughput gene expression experiment by several orders of magnitude [44]. 

Moreover, GFP establishes the gene hierarchy of that region based on their Gene Commanding Height [37]. The topgene, termed Gene Master Regulator (GMR), is the gene whose strictly controlled expression level regulates the major functional pathways by coordinating the expressions of most of their genes. Therefore, altered expression of the GMR has the largest consequences for cell physiology and can be used to selectively suppress the phenotype it commands.

### 2.2. Prostate Tissues

This study is based on expression data obtained by profiling the primary tumor (hereafter denoted as region “A”), two secondary tumors (regions “B” and “C”), and the surrounding cancer-free tissue (region “N”) from a prostate of a 65 year old black man. Raw and processed data from the four regions were deposited and are publicly accessible in the websites [45] for the “N” and “C” regions, and [46] for the “A” and “B” regions. The patient underwent a robotically assisted radical prostatectomy. The primary tumor had a Gleason score of 4 + 5 = 9/10 and the two secondary tumors had both the same Gleason score 4 + 4 = 8/10. The primary nodule was situated in the center of the left posterior quadrant, extending from the apex to the base. Both secondary nodules were situated right anterior mid-gland. The patient presented seminal vesicle invasion and metastasis in one right pelvic and iliac lymph node.

Each of the 6–8 mm samples collected from the four regions was split into four parts and each quarter was profiled separately, providing the needed four biological replicas. Although the selected regions were as homogeneous as possible, cells of different phenotypes were not completely eliminated, and by consequence, expression of genes from other cell phenotypes affected (diluted) the reported results. 

### 2.3. Microarray

At the time, we had equal access to Illumina NextSeq 500 but we preferred to use Agilent 4 × 44 k human dual-color microarrays (configuration G2519F, platform GPL13497 [47]) for their excellent reliability and affordable price. We used our standard protocol [44] for the RNA extraction with RNAEasy Minikit (Qiagen, Germantown, MD, USA), purification, and quantification before and after reverse transcription in the presence of Cy3/Cy5 dUTP with a Thermo Fisher Scientific NanoDrop ND-1000 (Waltham, MA, USA). RNA quality was checked with a 2100 Bioanalyzer (Santa Clara, CA, USA). 825 ng of differently (Cy3/Cy5) labeled biological replicas of the same prostate region were hybridized 17 h at 65 °C with microarrays and the washed and dried chips were scanned with an Agilent G2539 dual laser scanned for 20 bit at 5 µm resolution. The digital images (tiffs) were primarily analyzed with (Agilent) Feature Extraction vs. 11.6 software. The spots with saturated or corrupted pixels and those with the fluorescence foreground less than twice the fluorescence background were eliminated from the analysis. We used our iterative procedure alternating intra-array and inter-arrays adjustment to normalize the raw data to the median background subtracted fluorescence of all spots [48]. The control spots of the microarrays were used as technical replicas to estimate the technical noise of the method.

### 2.4. Transcriptomic Analyses

Agilent microarrays probe some genes redundantly with several (not uniform numbers of) spots; for instance, *TP53* was probed by 11 spots, all “transcript variant 1”. The independent characteristics of every gene across biological replicas: average expression level (AVE), Relative Expression Variability (REV), and correlation (COR) with an expression of other genes were determined using the expression levels of all valid spots probing that gene in “*region*” (= “N”, “A”. “B”, “C”) as:

(1) $$AVE_{i}^{\left(region\right)}=\frac{1}{R_{i}}\overset{R_{i}}{\underset{k=1}{\sum }}\mu_{i,k}^{\left(region\right)}=\frac{1}{R_{i}}\overset{R_{i}}{\underset{k=1}{\sum }}\left(\frac{1}{4}\overset{4}{\underset{j=1}{\sum }}a_{i,k,j}^{\left(region\right)}\right)$$

where: “*R_i_*” is the number of spots probing redundantly gene “*i*”, $a_{i,k,j}^{\left(region\right)}$ is the expression of gene “i” probed by spot ”*k*” on biological replica “*j*” in “*region*”

(2) $$REV_{i}^{\left(region\right)}=\underset{\mathrm{correction}\;\mathrm{coefficient}}{\underset{︸}{\frac{1}{2}\left(\sqrt{\frac{r_{i}}{\chi^{2}\left(r_{i};0.975\right)}}+\sqrt{\frac{r_{i}}{\chi^{2}\left(r_{i};0.025\right)}}\right)}}\sqrt{\underset{\mathrm{pooled}\;\mathrm{CV}}{\underset{︸}{\frac{1}{R_{i}}\overset{R_{i}}{\underset{k=1}{\sum }}{\left(\frac{s_{ik}^{\left(region\right)}}{\mu_{ik}^{\left(region\right)}}\right)}^{2}}}}\times 100\%$$

where: “$\mu_{ik}$” is the average expression of and “$s_{ik}$” is the standard deviation of gene “*i*” probed by spot “*k*” in the four biological replicas of “*region*”. “*r_i_* = 4*R_i_* − 1” is the number of degrees of freedom.

(3) $$COR_{ig}^{\left(region\right)}=\frac{\sum_{k_{i}=1}^{R_{i}}\sum_{k_{g}=1}^{Rg}\left(\sum_{j=1}^{4}\left(a_{i,k,j}^{\left(region\right)}-AVE_{i}^{\left(region\right)}\right)\left(a_{g,k,j}^{\left(region\right)}-AVE_{g}^{\left(region\right)}\right)\right)}{\sqrt{\sum_{k_{i}=1}^{R_{i}}\left(\sum_{j=1}^{4}{\left(a_{i,k,j}^{\left(region\right)}-AVE_{i}^{\left(region\right)}\right)}^{2}\right)\sum_{k_{g}=1}^{R_{g}}\left(\sum_{j=1}^{4}{\left(a_{g,k,j}^{\left(region\right)}-AVE_{g}^{\left(region\right)}\right)}^{2}\right)}}$$

where “*g*” is another gene.

REV and COR are used to determine the Gene Commanding Height (GCH) [37]:

(4) $$GCH_{i}^{\left(region\right)}=\underset{\mathrm{transcription}\;\mathrm{control}\;\mathrm{estimate}}{\underset{︸}{\frac{{\langle REV\rangle }^{\left(region\right)}}{REV_{i}^{\left(region\right)}}}}\times \underset{\mathrm{measure}\;\mathrm{of}\;\mathrm{expression}\;\mathrm{coordination}}{\underset{︸}{\exp\left(4{\left.\bar{{\left(COR_{ij}^{\left(region\right)}\right)}^{2}}\right|}_{\forall j\neq i}\right)}},$$

where 〈 〉 = median, $\bar{{\left(\right)}^{2}}$ = average of the square values.

A gene was considered as statistically (*p* < 0.05) significantly regulated in a cancer nodule (“cancer”) with respect to the normal tissue if the absolute fold-change *x* and the *p*-value ($p_{i}^{\left(N\to cancer\right)}$) of the heteroscedastic *t*-test of the mean equality in the two regions satisfy the composite criterion:

(5) xi(N→cancer)>CUTi(N→cancer)=1+11002REVi(N)2+REVi(cancer)2 ^ pi(N→cancer)<0.05where:cancer=“A”,“B”,“C”xi(N→cancer)≡μi(cancer)μi(N), if μi(cancer)>μi(N)−μi(N)μi(cancer), if μi(cancer)<μi(N) , μi(N/cancer)=1Ri∑k=1Riμik(N/cancer)

The *p*-value was computed with Bonferroni correction for multiple testing [49] in the case of several spots probing redundantly the same gene. 

For more comprehensive characterization of the expression alteration, we computed the Weighted Individual (gene) Regulation (WIR) and, for an entire functional pathway Γ, the Weighted Pathway Regulation (WPR) [30] as

(6) $$\begin{array}{l} WIR_{i}^{\left(N\to cancer\right)}=AVE_{i}^{\left(N\right)}\frac{x_{i}^{\left(N\to cancer\right)}}{\left|x_{i}^{\left(N\to cancer\right)}\right|}\left(\left|x_{i}^{\left(N\to cancer\right)}\right|-1\right)\left(1-p_{i}^{\left(N\to cancer\right)}\right)\\ WPR_{\Gamma }^{\left(N\to cancer\right)}=\bar{{\left.WIR_{i}^{\left(N\to cancer\right)}\right|}_{i\in \Gamma }} \end{array}$$

Our software to determine these characteristics from the raw data is described in [50].

### 2.5. Pathway Analyses

In addition to the prostate cancer pathway (PRC), GFP was used here to analyze the KEGG-determined apoptosis (APO) [51], P53 signaling (P53) [52], and the (general) pathways in cancer (PAC) [53]. Within PAC, special attention was given to the gene blocks responsible for evading apoptosis and immortality (hereafter denoted by EAI, 46 genes), proliferation, insensitivity to antigrowth signals and block of differentiation (PIB, 54 genes), and resistance to chemotherapy and sustained angiogenesis (RCSA, 19 genes).

## 3. Results

### 3.1. Overview

In total, we quantified the expressions of 14,908 unigenes in each of the 16 quarters of the three cancer nodules (“A”, “B”, “C”) and the surrounding normal tissue (“N”), isolated from a surgically removed metastatic prostate tumor. The 4-biological replicas strategy provided for every gene in each region the values of AVE and REV. AVE was used to identify up-/down regulated genes in the “A”, “B”, “C” regions with respect to “N” and the differentially expressed genes between pairs of cancer nodules. REV analysis identified the very stably expressed genes (low REVs), critical for the survival and proliferation of each cell phenotype, and the very unstably expressed genes, used by the cells as vectors of adaptation to the environmental fluctuations [54]. Moreover, quantification of tens of thousands of genes at a time from the same region provided for each gene extra 14.907 correlation coefficients (COR) with each other gene within one region and 3 × 14,908 correlations with all genes from each other region. Thus, the use of GFP translated the quantified 56,632 expression values (4 regions × 14,908 genes in one region) data into 1,778,077,160 transcriptomic characteristics of the profiled tumor (119,270 times larger than the number of expression levels of the individual genes considered by the traditional analysis). 

The smallest and the largest AVEs in the four regions (multiples of the median gene AVE) were for “N”: bradykinin receptor B1 (*BDKRB1*; 0.11) and ribosomal protein L13 (*RPL13*; 621.27), for “A”: glycoprotein A33 (*GPA33*; 0.15) and *RPL13*; (288.47), for “B”: lymphocyte antigen 6 complex, locus G6C (*LY6G6C*; 0.07) and *RPL13* (476.68); for “C”: ubiquitously transcribed tetratricopeptide repeat-containing, Y-linked (*UTY*; 0.09) and *RPL13* (415.06). *RPL13,* the gene with the largest expression in all four regions 621.27 in “N”, 288.47 in “A”, 476.68 in “B” and 415.06 in “C”), has also extraribosomal functions, being involved in several diseases, including the gastric, colorectal and hepatic cancers [55]. Interestingly, more than half of the 50 most largely expressed genes in each region encoded ribosomal proteins: 26 in “N”, 31 in “A”, 37 in “B”, and 33 in “C”. The preferred down-regulation of the ribosomal proteins was somehow compensated by the preferred up-regulation of the polymerase subunits and several translation initiation factors. 

The most stably expressed (lowest REV) and the most unstably expressed (highest REV) genes in the four regions were for “N”: mitochondrial ribosomal protein S12 (*MRPS12*; 0.32%) and ubiquitously-expressed, prefoldin-like chaperone (*UXT*; 133.35%), for “A”: ectonucleoside triphosphate diphosphohydrolase 2 (*ENTPD2*; 0.29%) and ubiquitin-specific peptidase 31 (*USP31*; 188.14%), for “B”: synovial sarcoma, X breakpoint 3 (*SSX3*; 0.94%) and v-maf avian musculoaponeurotic fibrosarcoma oncogene homolog K (*MAFK*; 188.56%), for “C”: BAI1-associated protein 2-like 1 (*BAIAP2L1;* 0.40%) and zyxin (*ZYX*; 192.35%). Interestingly, in region “B”, both the most stably expressed (*SSX3*) and the most unstably expressed (*MAFK*) genes have recognized roles in various forms of cancers (e.g., [56,57]). 

The median REVs for all quantified genes are 12.79% (“N”), 32.39% (A), 29.73% (“B”), and 17.71% (“C”) indicating that the gene expressions are overall less controlled by the homeostatic mechanisms in the cancer regions. In thermodynamics, systems closer to equilibrium exhibit higher numbers of degrees of freedom (and by consequence larger variability measured by their entropy) and are more robust. We speculate that the overall REV hierarchy justifies why the cancer nodules are more robust systems (particularly the primary tumor “A”), with higher survival and proliferation rates than the normal tissue.

### 3.2. Independent Characteristics of the Individual Genes

**Figure 1:**
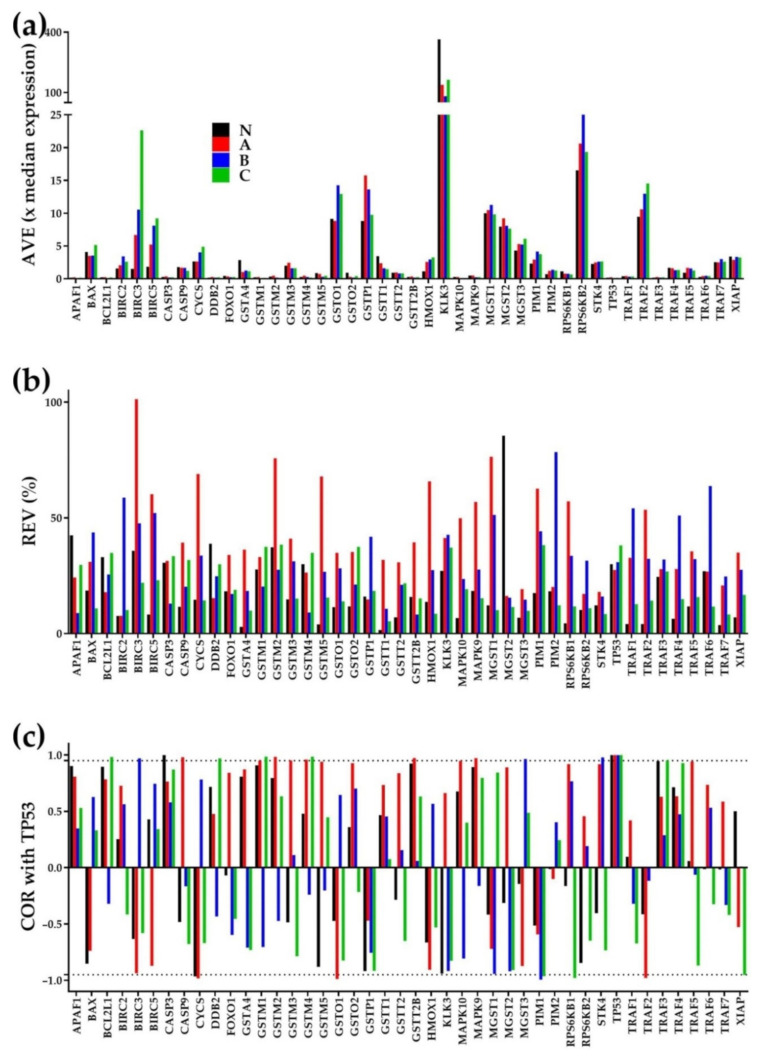
Three independent characteristics for every of the KEGG-determined 43 evading apoptosis genes in the three cancer nodule regions (“A”, “B”, “C”) and the surrounding normal tissue (“N”) region. (**a**) Average expression level (AVE in multiples of the average expression level of the median gene). (**b**) Relative Expression Variability. (**c**) Expression correlation with TP53.

Figure 1 presents the visual proof that for every gene in each region, AVE, REV, and COR (with each other gene from the same or another region) are independent characteristics. The independence is illustrated for the AVEs and REVs of 44 KEGG-selected evading apoptosis genes from the Pathways in cancer [44] and their expression correlation (COR) with *TP53*. The Pearson correlation coefficients among these three characteristics in each region were between −0.00069 and 0.00123, which is within the statistically significant independence interval. 

In this gene selection, kallikrein-related peptidase 3 (*KLK3*) had by far the largest average expression level (measured in median gene average expression level units) in all regions (364 in “N”, 139 in “A”, 82 in “B” and 164 in “C”). *KLK3* is a prognostic marker for progression-free survival in patients with metastatic prostate cancer [58]. However, *KLK3* has not the largest REVs, nor the highest correlation coefficients with *TP53* in any of the regions, verifying the independence of the three characteristics.

Microsomal glutathione S-transferase 2 (*MGST2*) had the largest variability in “N” (REV = 86%), baculoviral IAP repeat containing 3 (*BIRC3*) had the largest variability in “A” (REV = 101), Pim-2 proto-oncogene, serine/threonine kinase (*PIM2*) in “B” (REV = 79%) and glutathione S-transferase mu 2 (*GSTM2*) in “C” (REV = 39%). *MGST2* was recently reported as critical in aristolochic acid-induced gastric tumor process [59]. *BIRC3* [60] and *PIM2* [61] are recognized anti-apoptotic factors, and *GSTM2* is a biomarker for the early stages of the prostate cancer [62].

COR analysis, validated by the unit values of the correlation of TP53 with itself in all four regions, indicates also different levels of synergism/antagonisms both across the regions for the same gene and across the genes within the same region. Expression correlations of two genes can be also opposite in different regions, indicating that the encoded products of the two genes act synergistically in one region and antagonistically in the other. For instance, BIRC3 has a (*p* < 0.05) significant antagonism with TP53 in “A” but a significant synergism in “B” (and a not significant antagonism in “C”.

Comparing the three characteristics of the same gene in the cancer regions with the normal tissue reveals that in addition to the expression level, cancer may alter also the control of the transcript abundance and expression coordination with other genes. Alteration of the expression control and/or coordination may occur for both regulated (e.g., *BIRC3*) and not regulated (e.g., *MGTS2*) genes. 

Figure 1 shows also the substantial differences among the three cancer nodules with respect to each of the three independent variables, indicating that the recognized tumor transcriptomic heterogeneity [34,35,36] does not stop at the expression levels of genes in the four regions. Tumor heterogeneity in the control of transcripts abundances and gene networking indicates distinct alterations of the cellular biological processes and remodeling of the functional pathways as illustrated below.

### 3.3. Three Ways to Measure the Expression Regulation

The most down-regulated genes were: high mobility group AT-hook 2 (*HMGA2*; −15.88× in “A”), kallikrein-related peptidase 11 (*KLK11*; –57.82× in “B”), and peptidase inhibitor 15 (*PI15*; −30.20× in “C”). The most up-regulated genes were forkhead box J1 (*FOXJ1*; 13.75× in “A”), phospholipase A2, group IIA (platelets, synovial fluid) (*PLA2G2A*; 18.75x in “B”), and scleraxis basic helix-loop-helix transcription factor (*SCX*; 300.17× in “C”). The high up-regulation of *SCX* in “C” was a surprise given that its expression level was not altered in the other two cancer regions.

**Figure 2:**
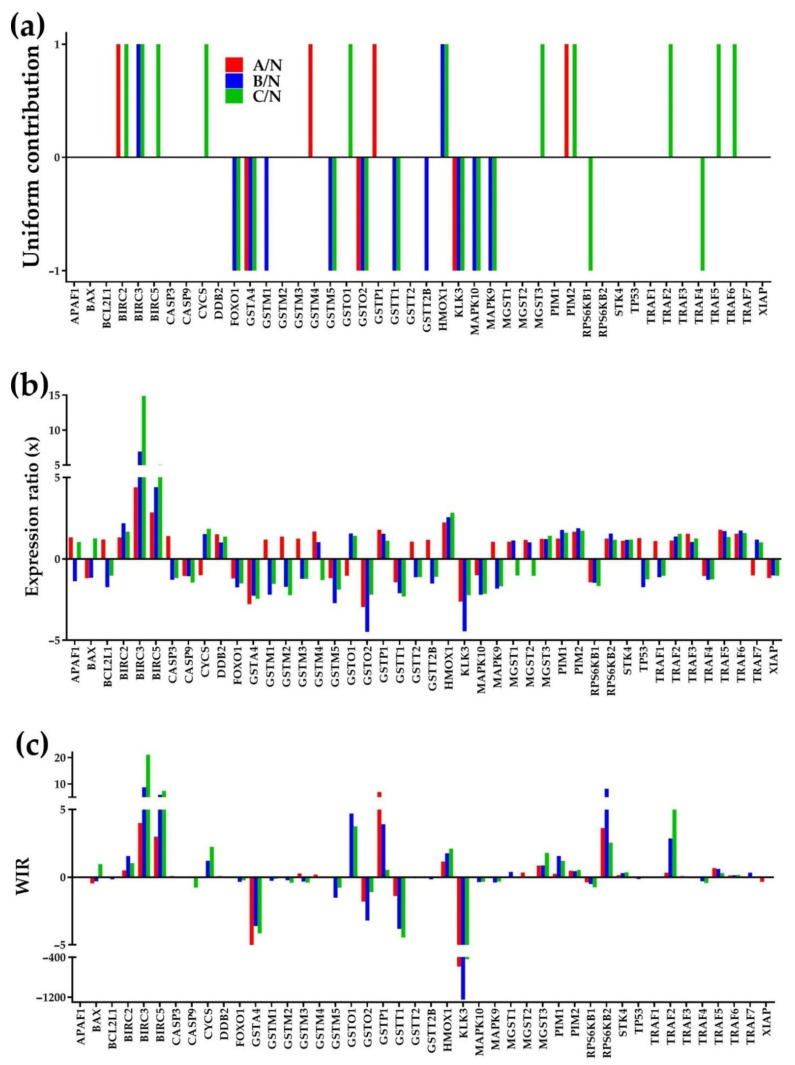
Regulation of 44 evading apoptosis genes in the three cancer nodules (“A”, “B”, “C”) with respect to the normal tissue (N) measured as (**a**) uniform contribution of the significantly regulated genes, (**b**) expression ratio “x” and (**c**) Weighted Individual (gene) Regulation “WIR”.

Figure 2 presents the regulation of the KEGG-determined 44 evading apoptosis genes in the three cancer nodules (“A”, “B”, “C”) with respect to the surrounding normal tissue (“N”). The regulation is shown as Figure 2a uniform (+1/−1) contribution to the percentages of up-/down-regulated genes, Figure 2b expression ratio “*x*” and Figure 2c Weighted Individual (gene) Regulation (“*WIR*”). Both *x* and *WIR* are negative for down-regulated genes. 

While the “uniform contribution” is limited to only significantly regulated genes based on arbitrary cut-off criteria for the absolute fold-change and the p-value of the heteroscedastic *t*-test of the means’ equality, “*x*” and “WIR” considers all genes and discriminate their contributions. For instance, although the up-regulation of *BIRC3* had the same contribution (+1) to the percentage of up-regulated genes in both “B” and “C” compared to “N”, the expression ratio in “C” (*x* = 14.89) is statistically significantly larger than in “B” (*x* = 6.94) and so is the WIR (21.05 in “C” and 8.71 in “B”). However, although in this gene selection, the expression ratio with respect to “N” was the largest for *BIRC3* in “C”, it is the down-regulation of *KLK3* that had the largest absolute contribution in all cancer nodules (WIR = −585.50 in “A”, −1,248.43 in “B” and −439.17 in “C”) to the transcriptomic alteration in all cancer nodules “B”, owing to its large expression level in the reference tissue (364 in region “N”).

### 3.4. Regulation of Selected Functional Pathways in the Cancer Nodules with Respect to the Normal Tissue

**Figure 3:**
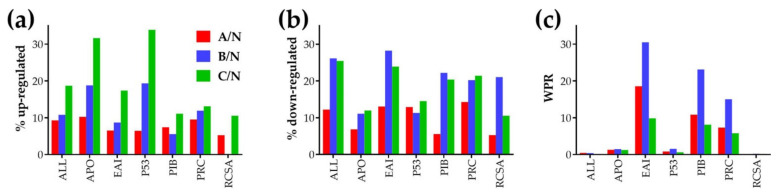
Overall regulation of selected KEGG-determined functional pathways measured by the percentages of the significantly down- (**a**) and up-regulated (**b**) genes, and by the Weighted Pathway Regulation (WPR) (**c**). ALL = all genes, APO = apoptosis, EAI = evading apoptosis + immortality, P53 = P53 signaling, PIB = proliferation + insensitivity to antigrowth signals + block of differentiation, PRC = prostate cancer, RCSA = resistance to chemotherapy and sustained angiogenesis.

Figure 3 presents the regulation of the major KEGG-determined functional pathways: APO (117 quantified genes), P53 (62 genes), PRC (84), and selected component blocks of PAC: EAI (46 genes), PIB (54 genes), RCSA (19 genes). The pathway regulations were quantified as percentages of up- and down-regulated genes and as Weighted Pathway Regulation (WPR). For comparison, we added also the numbers when all (ALL) 14,908 quantified genes are considered. Of note is that nodule B had the significantly largest WPRs for the EAI, PIB, and PRC groups of genes, while nodule C had the significantly largest percentages of up-regulated genes in all pathways. Of note is also the finding that in all cancer nodules more genes related to cancer cell survival and proliferation are up- than down-regulated, while more genes involved in apoptosis and P53 signaling are down-regulated than up-regulated.

### 3.5. Differential Regulation of the Genes Central to the KEGG-Determined Prostate Cancer Pathway in the Three Cancer Nodules

**Figure 4:**
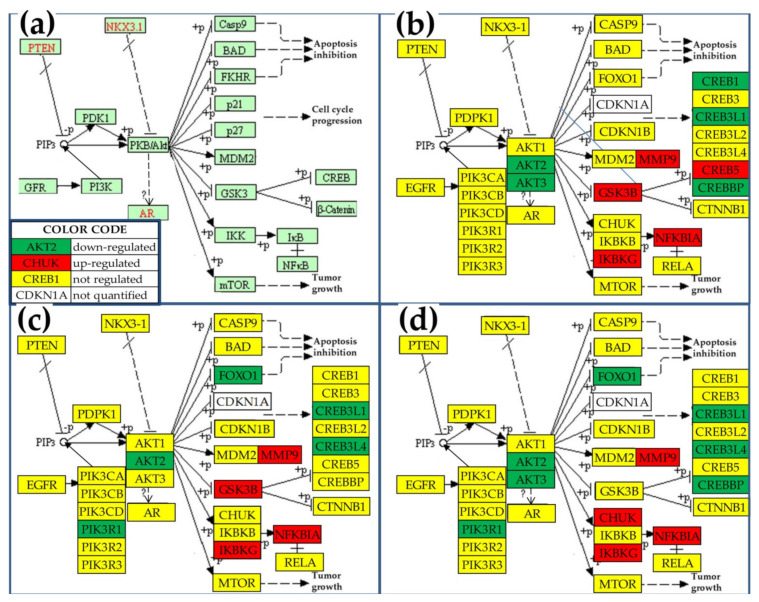
Significantly regulated genes central to the (**a**) KEGG-determined prostate cancer functional pathway in the: (**b**) nodule “A”, (**c**), nodule “B”, (**d**) nodule “C” with respect to the surrounding normal tissue (“N”). Regulated genes: *AKT2/3* (v-akt murine thymoma viral oncogene homolog 2/3), *CREB1/5* (cAMP-responsive element-binding protein 1/5), *CREB3L1/4* (cAMP-responsive element-binding protein 3-like 1/4), *CREBBP* (CREB binding protein), *FOXO1* (forkhead box O1), *GSK3B* (glycogen synthase kinase 3 beta), *IKBKG* (inhibitor of kappa light polypeptide gene enhancer in B-cells, kinase gamma), *MMP9* (matrix metallopeptidase 9 (gelatinase B)), *NFKBIA* (nuclear factor of kappa light polypeptide gene enhancer in B-cells inhibitor, alpha), *PIK3R1* (phosphoinositide-3-kinase, regulatory subunit 1 (alpha)). (modified from [39]).

Figure 4 shows the regulation of individual genes from the blocks of genes (presented in Figure 4a) at the center of the KEGG-determined prostate cancer pathway [39]. Genes shown in red/green background exhibited significant regulation in the cancer region with respect to the normal tissue, while genes shown in the yellow background did not satisfy our composite criterion (5). However, though not significant, owing to the biological variability, technical noise of the method, and the stochastic nature of the gene transcription, the average expression levels of the genes in the yellow background are most likely not identical in the compared regions. Although not very abundant, there are still significant differences in the subsets of the regulated genes among the three nodules. The up-regulation of *MMP9* (matrix metallopeptidase 9 (gelatinase B) is in line with the reported higher expression in the serum of patients with lung [63] and prostate [64] cancer. Although *AKT2* (v-akt murine thymoma viral oncogene homolog 2) was down-regulated in all three cancer nodules (*x* = −1.65 in “A”, −1.61 in “B” and −1.94 in “C”), AR (androgen receptor) was not affected and therefore the tumor did not regress as expected in the androgen-deprivation therapy [65].

### 3.6. Regulation of Individual Genes Responsible for Survival and Proliferation of Cancer Cells

Figure S1 in the Supplementary Materials present the expression regulation of genes identified by KEGG [47] as linked in most cancer forms to the: block of differentiation, evading apoptosis, immortality, insensitivity to anti-growth signals, proliferation, resistance to chemotherapy, and sustained angiogenesis. Genes such are *GSTA4, GSTO2, KLK3, PGF* were down-regulated and *E2F2, MMP9, PIM2, VEGFC* are up-regulated in all cancer nodules. Other genes were regulated only in one nodule, e.g., *CSF1R, CYCS, E2F3, GSTM1, GSTM4*, *GSTO1, GSTP1, GSTT2B, HES1, HEY1, HEYL, HHIP, MGST3, MYC, RARA.* There are also genes regulated similarly in two nodules: *BIRC2, BIRC3, BMP4, CCNA2, CCND1, E2F1, FOXO1*, *GLI1, GSTM5, GSTT1*. However, no gene was found as oppositely regulated in two of the three profiled cancer nodules. Of note are the differences in the subsets of significantly regulated genes among the three cancer nodules.

### 3.7. Remodeling of the Transcriptomic Networks

In addition to regulating the expression levels of numerous genes and altering the control of their transcripts’ abundances, cancer affects also the coordination of genes’ expressions, reconfiguring their networking in functional pathways, with direct consequences on cell biology. 

**Figure 5:**
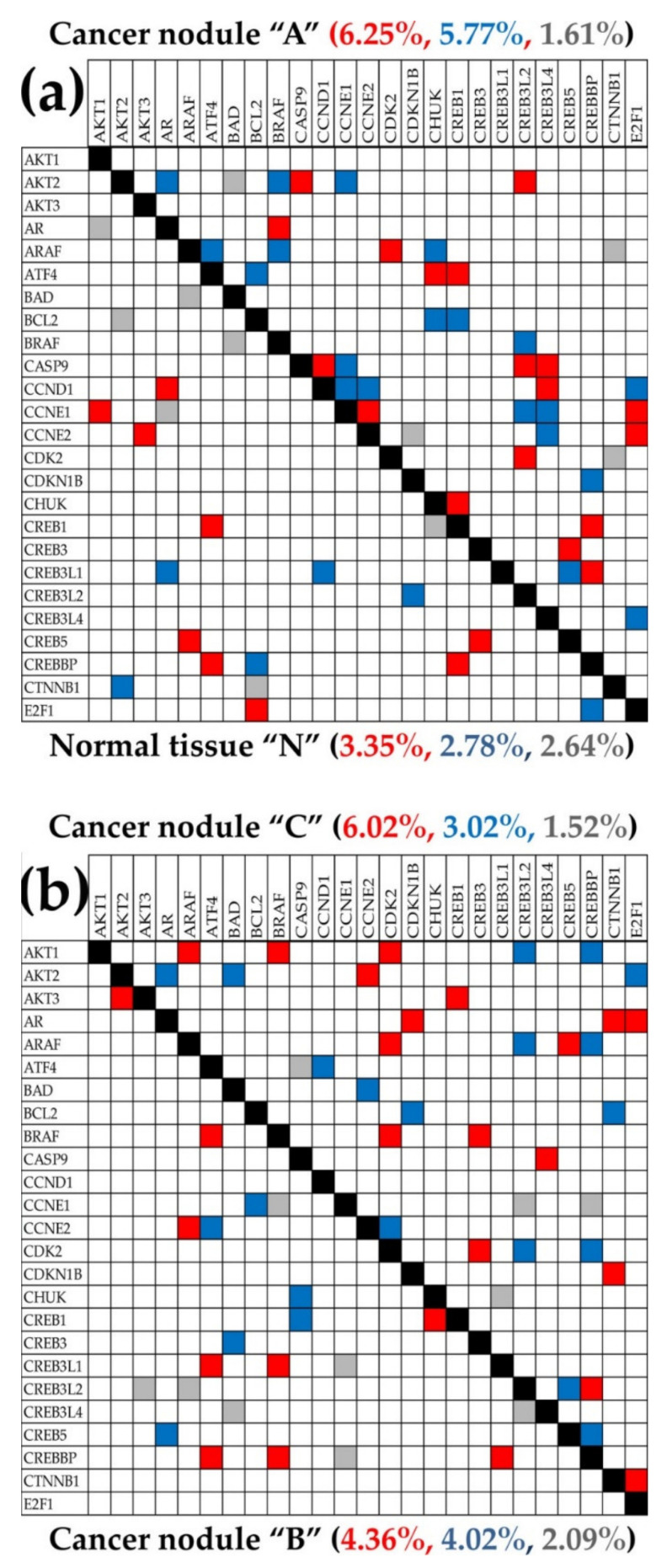
Statistically (*p* < 0.05) significant expression synergism, antagonism, and independence of the first 25 alphabetically ordered KEGG-identified prostate cancer-related genes in the normal tissue (N) (**a**) and each of the three cancer nodules (“A” (**a**), “B”, “C” (**b**)). Red/blue/gray squares indicate significant expression synergism/antagonism/independence of the genes labeling the intersecting rows and columns in that profiled region. Blank squares specify less than significant statistical evidence to categorize the corresponding gene pairing. Red/blue/gray numbers are the percentages of significant expression synergism/antagonism/independence among the first 25 alphabetically ordered prostate cancer genes quantified in that region. Owing to the correlation analysis symmetry to the permutation of the correlated genes, only the below or over the diagonal (black squares) part of the diagram was shown for each condition.

Figure 5 illustrates the remodeling of the gene networks by presenting the statistically (*p* < 0.05) significant expression synergism, antagonism, and independence of the first alphabetically ordered 25 out of the 84 quantified KEGG-identified prostate cancer-related genes [39] in the normal tissue and each of the cancer nodules. 

One may observe that each cancer nodule had higher expression (synergistic + antagonistic) coordination and lower independence than the normal tissue, indicating stronger inter-coordination of the prostate cancer genes in the cancer nodules. Thus, 12.02% total coordination in “A” > 9.04% total coordination in “C” > 8.38% total coordination in “B” > 6.13% total coordination in “N”. Of note are also the coordination differences among the three cancer nodules.

### 3.8. Alteration of the TP53 Targeted Genes Network

In the above paragraph, we proved that cancer changes the expression coordination among genes, thus rearranging the gene’s transcriptomic interlinkages. Remodeling of the gene networks affects the activation/inhibition of targeted genes by hub genes, with major consequences on the dynamics of the cellular processes. 

Figure S2 in the Supplementary Materials presents the correlation networks by which *TP53* coordinates the expression of several genes in the normal tissue and how these coordinations were changed in the three cancer regions. According to the KEGG-determined P53 signaling pathway [46], all genes from all blocks of genes presented in Figure 7 should be activated by *TP53*. In terms of expression coordination, it means that expression of the target genes should be synergistically expressed with that of *TP53* (i.e., up when *TP53* goes up and down when *TP53* goes down). However, only part of the targeted genes was actually synergistically expressed with *TP53* in the normal prostate tissue (*GTSE1, SIAH1 SESN2, SESN3 RCHY1, PPMID*) and one (*SIVA1*) even antagonistically expressed. The expression correlations in the normal tissue were substantially (but differently) altered by cancer in the three nodules. The numbers of correlations ranged from 1 synergism and 3 antagonisms in “B” to 5 synergisms and 2 antagonisms in “A”, and 4 synergisms and 5 antagonisms in “C”. The subsets of significantly regulated genes were also different in the three cancer nodules: 4 up- and 6 down-regulated in “A”, 5 up- and 3 down-regulated in “B”, and 13 up- and 4 down-regulated in “C”.

Figure S2 shows also what genes activated by *TP53* were up/down-regulated by cancer. One may note that not only the expression coordinations with *TP53* were different among the cancer nodules but also the expression regulation of the target genes.

Alteration of TP53 (e.g., [66]) and another signaling (e.g., [67]) pathways in the prostate cancer were analyzed by several other groups before, but this is the first time after our knowledge that the analysis goes beyond expression regulation to explore also the expression coordination.

### 3.9. In-Phase and in Anti-Phase Expression of Prostate Cancer Genes among the Three Cancer Nodules

Figure S3 presents the KEGG-identified prostate cancer genes [39] that are statistically (*p* < 0.05) significantly expressed in-phase and in anti-phase among the profiled regions of the tumor. This analysis is based on our model of transcellular transcriptomic networks [43] that was also recently applied to identify synchronous expression of ion channels between adjacent heart chambers [68].

One may note that the cancer nodules have different in-phase and anti-phase expression correlations of prostate cancer genes both among themselves and with respect to the normal tissue region, nodules “A” and “B” having the largest in-phase expressions. Interestingly, while region “C” has the least in-phase and in anti-phase expressions with both regions “A” and “B”, it has the highest number of in-phase expressions with the normal tissue. We found that *ERBB2* (v-erb-b2 avian erythroblastic leukemia viral oncogene homolog 2, a.k.a., *HER2*) is expressed in-antiphase in “N” with respect to nodules “A” and “B” but in-phase between these two nodules. This finding justifies the reports that *ERBB2* activation is associated with tumor-initiating cells contribution and progression of prostate cancer [69].

### 3.10. Gene Hierarchy

**Figure 6:**
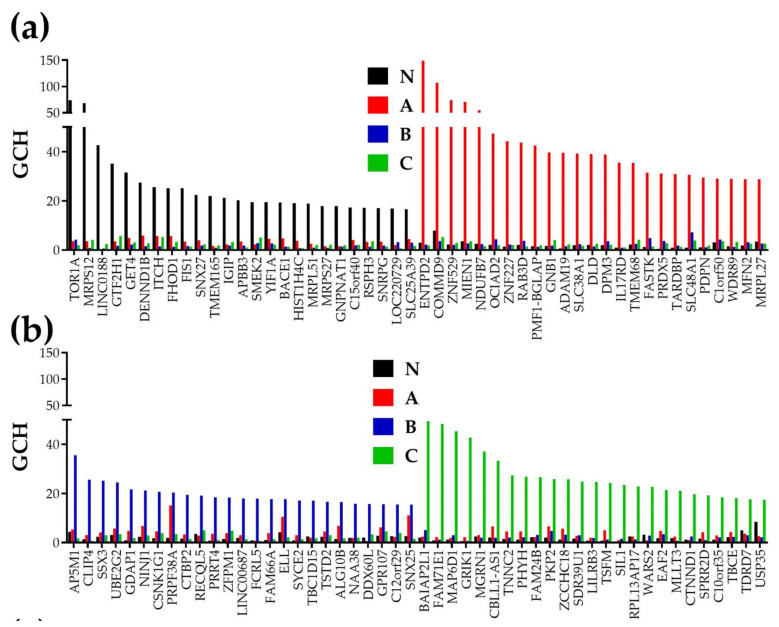
Top 25 genes in each region and what may happen to the regulated enzyme genes in “A” by overexpressing *ENTPD2*, the GMR of “A”. (**a**) Top 25 genes in “N” and “A”; (**b**) Top 25 genes in “B” and “C”.

Figure 6 presents the top 25 genes in each of the four profiled regions. The gene hierarchies were determined separately for each profiled region based on their GCH score (defined by the formula (4)) and the top of the top genes (the GMR) identified in each region. 

The very important results of the GCH analysis are: (i) there is no overlap of the top 25 genes among the profiled regions; (ii) The GMR of one region has significantly lower GCH scores in the other regions; (iii) top genes may include both coding and non-coding genes. 

The GMRs are: *TOR1A* (torsin family 1, member A), GCH = 74 in “N”, *ENTPD2* (ectonucleoside triphosphate diphosphohydrolase 2), GCH = 149 in “A”*, AP5M1* (adaptor-related protein complex 5, mu 1 subunit) GCH = 36 in “B” and *BAIAP2L1* (BAI1-associated protein 2-like 1), GCH = 49 in “C”. 

The non-coding genes within the top 25 are *LINC0188* (long intergenic non-protein coding RNA 1881), *C15orf40* (chromosome 15 open reading frame 40), and *LOC220729* (succinate dehydrogenase complex, subunit A, flavoprotein (Fp) pseudogene) in “N”, *C12orf29* (chromosome 12 open reading frame 29) in “A”, *LINC00687* (long intergenic non-protein coding RNA 687) and *C12orf29* (chromosome 12 open reading frame 29) in “B”, and *RPL13AP17* (ribosomal protein L13a pseudogene 17) in “C”.

The top ranking of *TOR1A* in the normal prostatic tissue justifies its major roles in protein folding, degradation and trafficking, vesicle fusion, cytoskeleton dynamics, organelle biogenesis, and spermatogenesis [70]. We found *TOR1A* as significantly up-regulated in the nodule “B” (*x* = 1.44), but not altered in the other two cancer nodules. 

A higher GCH score indicates the stronger regulatory power of the GMR on the phenotype it commands. With GCH = 149 in “A” but only 3 in “N” and 2 in both “B” and “C”, *ENTPD2* is a strong candidate target gene whose manipulation may selectively destroy the primary tumor “A” with practically no effect on the other regions of the prostate. Recent reports considered *ENTPD2* relevant for the lung adenocarcinoma [71] and hepatocellular carcinoma [72] transcriptomic signatures. Interestingly, expression of *ENTPD2* was significantly up-regulated in the nodules “B” (*x* = 2.95) and “C” (*x* = 2.03) but not in “A” (*x* = −1.08).

*AP5M1* (a.k.a. *MUDENG*), the GMR of nodule “B”, is already considered a target for anti-cancer therapy owing to its pro-apoptotic function [73]. Owing to its very low expression variability, the 20% increase of *AP5M1* expression level in the nodule “B” was statistically significant. However, its expression level was not significantly altered in the nodules “A” or “C”. 

*BAIAP2L1,* the GMR of nodule “C” was up-regulated in all three cancer regions (by 1.76× in “A”, 2.54× in “B”, and 1.92× in “C”). Up-regulation of *BAIAP2L1* was observed in clear cell Renal Carcinoma [74] (confirmed also by our data on a surgically removed ccRCC [75]), in lung cancer [76] and recently even in prostate cancer [77].

In all profiled regions, the top genes are of wide functional diversity, indicating a strong interplay of the functional pathways. For instance, the next two genes in “N” are: mitochondrial ribosomal protein S12 (*MRPS12*, GCH = 69) and *LINC0188* (GCH = 43), and the next genes in “A” are: COMM domain containing 9 (*COMMD9*, GCH = 107) and zinc finger protein 529 (ZNF529, GCH = 74). In addition to the GMRs, the commanding triplet in “B” includes CAP-GLY domain-containing linker protein family, member 4 (*CLIP4*, GCH = 26) and synovial sarcoma, X breakpoint 3 (*SSX3*, GCH = 25), while that of nodule “C” includes: family with sequence similarity 71, member E1 (*FAM71E1*, GCH = 48) and MAP6 domain containing 1 (*MAP6D1*, GCH = 45).

### 3.11. The GMR Approach of the Prostate Cancer Gene Therapy

Because *ENTPD2* is an enzyme (involved in the purine metabolism [78]), we analyzed its expression coordination with all other 3204 enzymes quantified in the nodule “A”, out of which 324 (10.11%) were up-regulated and 327 (10.21%) were down-regulated with respect to “N”. 311 (11.14%) enzyme genes were (*p* < 0.05) significantly coordinated (|COR| > 0.95) with *ENTPD2*: 102 (3.18%) synergistically expressed and 209 (7.96%) antagonistically. As we proved in previous works [43,79], genes synergistically expressed with the target gene tend to be up-regulated when the target is experimentally over-expressed while those antagonistically expressed tend to be down-regulated. 

**Figure 7:**
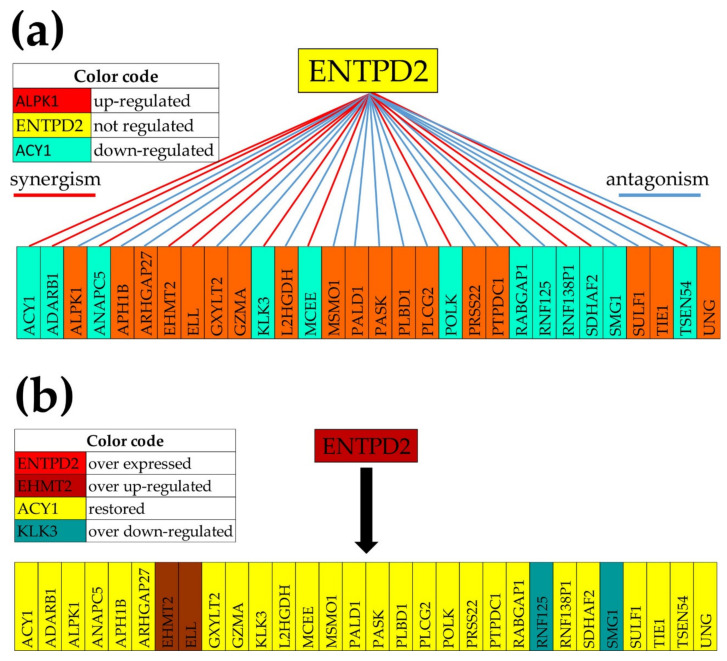
The GMR at work. (**a**) Significant expression coordination of the significantly regulated enzyme genes with *ENTPD2* in “A”. (**b**) Expected regulation of the enzymes following overexpression of *ENTPD2*. Note that all genes but *EHMT2* (euchromatic histone-lysine *N*-methyltransferase 2), *ELL* (elongation factor RNA polymerase II), *RNF125* (ring finger protein 125, E3 ubiquitin protein ligase), and *SMG1* (SMG1 phosphatidylinositol 3-kinase-related kinase) restored their normal expression.

Figure 7 presents the significant expression coordination of *ENTPD2* with significantly regulated enzyme genes in “A” and what might happen if *ENTPD2* is experimentally overexpressed. 

The analysis predicted that 16 out of 18 significantly up-regulated enzymes that were antagonistically expressed with *ENTPD2*, and 10 out of 12 significantly down-regulated enzymes synergistically expressed with *ENTPD2*, are expected to restore their normal expression after significantly overexpressing *ENTPD2.* We assume that this 26/30 (87%) restoration of the normal expression of the enzyme genes will force the cancer cells from the nodule “A” to go back to the normal phenotype. Owing to its very large (negative) contribution to the cancer phenotype in all three nodules (Figure 2c) and its antiangiogenic activity [80], restoration of the normal expression of *KLK3* may be instrumental for the normal phenotype recovery.

## 4. Discussion

Our work was not meant to produce results with general validity but to illustrate how the novel paradigm (GFP, [40,81]) can be applied to prostate cancer as a personalized alternative to the biomarker paradigm. GFP does not stop after identifying what genes were regulated by cancer but goes on to quantifying how much cancer changes the cellular control of transcripts’ abundances and remodels the gene networks. 

In addition to the inherent technical noise of the microarrays, the major limitation of our study comes from the morphological heterogeneity of the tumor reflected at the transcriptomic level [34,35,36]. Although we did our best to isolate the most homogeneous parts of the cancer nodules and normal tissue, the samples were not 100% pure. However, the use of scRNA-sequencing was not an option owing to the sparse returns. With almost 90% random “dropout” of the expressed genes [82], the use of scRNA-sequencing would have drastically limited the GFP analytical power. However, our algorithms are fully developed and ready to be used when the scRNA technological limitations will be solved.

The most important result is the identification of the Gene Master Regulators (GMR) of cancer nodules whose tightly controlled expressions impose the phenotype by coordinating the expressions of most other genes. As such, the GMRs command the cellular functional pathways, and their directed alteration might eliminate the cancer phenotype. Our analysis suggests that GMR targeting may enhance apoptosis (as in [79]), stop cell proliferation [37], significantly alter cell cycle [37], or force cancer cells to go back to the normal transcriptome as hypothesized in Section 3.11. Unfortunately, we could not functionally validate these predictions on the profiled tumors. Nonetheless, we validated the relationship between the GCH score and the transcriptomic consequences of manipulating the gene expression by stably transfecting *DDX19B*, *NEMP1*, *PANK2,* and *UBALD1* on the papillary (BCPAP) and anaplastic (850C) human thyroid cancer cell lines [51,53]. The cited studies confirmed that experimental changing the expression of a gene in the two cell lines produced larger alterations of the transcriptomic organization in the cells where that gene had higher GCH. 

The GFP approach provided the most theoretically possible comprehensive understanding of the transcriptomic topology. GFP characterizes each gene by the independent variables: average expression levels (AVE), relative expression variability (REV), and expression correlation (COR) with each other genes. To understand the importance of these characteristics let us think about what is needed to assemble an electronic device with tens of thousands of different types of semiconductors. Using the right numbers (AVE) of each type of electronic component but not applying the right voltage oscillations across these components (REV) would provide different outputs. The situation is worse if the wiring of the components (COR) is not respected. Likewise, knowing only the average expressions of the genes is not enough to understand cancer complex genomics.

The correlation analysis is popular in clustering the genes according to their similar regulation across people, conditions, tissues, or time-points (e.g., [83,84,85,86]). It was even used to correlate transcripts and protein abundances [87]. However, all our genomic studies indicated that the gene networking differs from person to person and from tissue to tissue, change with maturation/aging, remodel in disease and recovery following adequate treatment. Thus, although clustering is useful for classification, it does not reflect gene interactions. In contrast, our COR analysis restricted to biological replicas shows how the expression of one gene fluctuates in-phase/in anti-phase with expressions of other genes, a pre-requisite for optimal gene networking in functional pathways. 

The analyses indicated that the three cancer nodules had distinct transcriptomic organizations beyond the gene expression regulations (reported also by other authors [34,35,36]). Thus, we found different alterations of controls of transcripts’ abundances and, distinct remodeling of the gene networks even between the phenotypically similar regions “B” and “C”. 

These results prove again the existence of transcriptomic redundancy (many distinct transcriptomes) of each cancer phenotype that poses serious challenges to the use of disease “transcriptomic signatures” [88,89,90]. We have arrived at the same conclusion not only for different persons (equally graded cases of prostate cancer [30]) but even for equally graded cancer nodules from the same tumor (one case of clear cell renal cell carcinoma [37,91] and this case of prostate tumor). 

REV analysis pointed also to some interesting genes for oncogenomics. Thus, *MRPS12*, the most stably expressed gene in “N”, is a potential prognostic candidate for ovarian cancer [92], while *UXT* the most unstably expressed gene in “N” is associated with advanced stages of gastric cancer [93]. *ENTPD2*, the most stably expressed gene in “A” is a biomarker for lung [94] and hepatocellular [72] carcinomas, while *USP31*, the most variably expressed gene in “A”, could be a potential target for sarcomas [95]. *SSX3*, the most stably expressed in “B” is a prognostic predictor for pancreatic ductal adenocarcinoma [96], while *MAFK*, the most variably expressed gene in “B”, appears responsible for the induction of breast cancer [97]. *BAIAP2L1*, the most stably expressed gene in “C”, is overexpressed in clear cell renal cell carcinoma [74] as we also found in all three prostate cancer nodules, while *ZYX*, the most variably expressed gene in “C” and a crucial mechanotransductor in prostate cancer [98], had also a very large (176.65×) up-regulation in “C” (*ZYX* was also up-regulated in “A” by 2.53× and “B” by 2.92×). The significant difference in the fold-change of this gene between nodules “B” and “C” suggests distinct mechanotransduction mechanisms (worth being further investigated by molecular and physiological studies). The median REVs are 12.79% (“N”), 32.39% (“A”), 29.73% (“B”), and 17.71% (“C”) indicating that the gene expressions are overall less controlled by the homeostatic mechanisms in the cancer regions. 

Interestingly, *HMGA2*, the strongest down-regulated gene in “A” (−15.88×), “whose silencing promotes apoptosis and inhibits migration and invasion of prostate cancer cells” [99], was not regulated in “B” and “C”. With *KLK11* down-regulated by −7.37× in “A”, −57.82× in “B” and −27.11× in “C”, our study confirmed previous reports e.g., [100], of this gene down-regulation in cancer regions with respect to the surrounding benign tissue in prostate tumors. The observed up-regulation in all three cancer nodules of *FOXJ1* (13.75× in “A”, 18.51 in “B” and 20.39 in “C”) and *PLA2G2A* (6.68× in “A”, 18.75× in “B” and 23.61× in “C”) confirmed findings of other authors [101,102]. 

Much more comprehensive than the fold-change, WIR analysis identified the genes with the largest contributions to the cancer-related transcriptomic alterations. Thus, the main contributors in “A” were: zinc finger protein 865 (*ZNF865*) and immunoglobulin lambda-like polypeptide 5 (*IGLL5*), in “B”: spondin 2, extracellular matrix protein (*SPON2*) and *IGLL5*, while in “C” they were *SPON2* and midkine neurite growth-promoting factor 2 (*MDK*). *IGLL5*, the main positive contributor to both nodules “A” and “B”, was correlated with tumor-infiltrating immune cells in kidney cancer [103], suggesting an interesting study to determine whether it plays a similar role in prostate cancer. *SPON2* is considered a serum prostate cancer diagnostic biomarker [104]. *MDK* is considered one of the most reliable prognostics for short prostate cancer-specific survival [105]. For now, we have no info about the potential roles of *ZNF865* in the progression of prostate cancer.

As illustrated in Figure 1c and Figure S1, cancer remodels also the transcriptomic networks by which genes are linked to each other in functional pathways. Importantly, the remodeling was different from one cancer region to the other. These findings prove that the KEGG-designed functional pathways are not universally wired but are dependent on the cell phenotype and even the localization in the tumor. As such, the same manipulation of the expression of one gene may have different consequences on the other genes of the pathway in distinct phenotypes. (We arrived at the same conclusion when conditionally knocking down the *Gja1* gene encoding the gap junction channel protein Cx43 in the brains of two mouse strains [20] and the brain and heart of the same mouse). By consequence, efficient gene therapy for one person may not be as efficient for another person. Moreover, the treatment efficiency may be even different for distinct cancer nodules from the prostate of the same man.

Figure S2 brings another confirmation of the transcriptomic networks’ dependence not only on the cell phenotype (compare regions “N” and “A”), but also on the localization of that phenotype within the tumor (compare “B” and “C”) as we have reported before for the two atria and the two ventricles of the mouse heart [68]. We found that *TP53* activation of the target genes is substantially different among the four profiled regions of the tumor. The negative correlation of *TP53* and *SIVA1* in both normal tissue and the primary tumor “A” (Figure S2a), suggests that increased expression of *SIVA1* is reported to inhibit cervical cancer progression [106] (and hopefully also the progression of prostate cancer) may be achieved by down-regulating *TP53.* This possibility is based on our “see-saw” model [107] indicating that similar effects of the increase of one gene expression can be obtained either by up-regulating another gene with a similar coordination profile (the ordered set of Pearson correlation coefficients between the expression levels of that gene with each other gene) or by down-regulating one with opposite coordination. However, the correlation of *TP53* with *SIVA1* is poor in the other two cancer nodules, indicating that down-regulating *TP53* may have little consequences for *SIVA1* in the secondary tumors.

We analyzed also whether there is inter-regional transcriptomic communication by determining the expression correlation of KEGG-determined prostate cancer genes between two regions. A positive correlation means that the expression of that gene goes up and down simultaneously (in-phase) in both regions, while a negative correlation means that when the expression of one gene goes up in one region it goes down in the other (in-antiphase). In previous studies ([43,68,81]) we have provided experimental evidence about transcellular transcriptomic communication between astrocytes and oligodendrocytes in the brain and between the myocardial walls of the adjacent heart chambers of the mouse. This type of analysis may provide clues about the transcriptomic integration of heterogeneous tissues. 

Lastly, we determined the gene hierarchy in the four profiled regions of the tumor, identified the GMR of each region, and analyzed what might be the consequences of overexpressing *ENTPD2*, the GMR of the primary tumor nodule “A”. Out of the four GMRs, we focused on the *ENTPD2* because it has a significantly higher GCH in its region than the other GMRs in their commanded regions. With 11.14% (*p* < 0.05) and 22.97% (*p* < 0.10) significant coordination with enzyme genes, expression manipulation of *ENTPD2* should have devastating consequences on the metabolism of the cells of nodule “A”. We have to warrant that this is a personalized genomic result with little relevance for the prostate cancers of other persons.

The idea of master regulators is floating in genomics for a long time, most investigators looking for transcription factors (e.g., [108,109]) or even hormone receptors (e.g., [110]) whose regulation might have large downstream effects on the expression of many genes. Distinct from the traditional approach, our procedure does not restrict the GMR quest to transcription factors but it ranks with respect to GCH all quantifiable coding AND non-coding RNAs. As illustrated in Figure 7, the top 25 genes of the profiled prostate tumor regions include long intergenic non-protein coding RNAs, pseudogenes, and open reading frames of various chromosomes. Thus, our method can identify novel genomic factors governing the cancer phenotype. Moreover, the coordination analysis allows predicting the consequences of the GMR alteration (illustrated in Figure 7).

## 5. Conclusions

We found that, beyond regulating the expression levels of numerous genes, prostate cancer altered also the cellular homeostatic mechanisms that control the abundances of their transcripts and, more importantly, their networking in functional pathways. Interestingly, even equally histopathologically graded cancer nodules from the same tumor have different transcriptomic organizations that go beyond the recognized tumor heterogeneity of gene expression levels (e.g., [32,33,34]). This conclusion raises serious concerns about the validity of biomarkers identified through meta-analyses of large populations of cancer-stricken and cancer-free humans and poses new challenges for the germline genetic testing of prostate cancer patients [111]. 

As illustrated here for one case of prostate cancer, and in previous papers on cases of papillary thyroid cancer [46,92] and clear cell renal cell carcinoma [35,93], GFP offers a comprehensive personalized alternative to the biomarkers approach to determine the cancer transcriptional identity (e.g., [112]) and explore gene therapy. 

## Data Availability

Raw and processed gene expression data were deposited and are publicly accessible at https://www.ncbi.nlm.nih.gov/geo/query/acc.cgi?&acc=GSE133906 (accessed on 14 April 2021) and https://www.ncbi.nlm.nih.gov/geo/query/acc.cgi?acc=GSE16871 (accessed on 14 April 2021).

## Supplementary Materials

The following are available online at https://www.mdpi.com/article/10.3390/cells10071644/s1, Figure S1. Expression coordination of TP53 with KEGG-determined targeted genes in (a) normal tissue; (b) cancer nodule “A”; (c) cancer nodule “B”; (d) cancer nodule “C”. Figure S2. Statistically (*p* < 0.05) significantly in-phase (synchronously) and in-antiphase expressed KEGG-determined prostate cancer genes in pairs of profiled regions.
