# Supplementary material for: A Personalized Genomics Approach of the Prostate Cancer
Source: Cells. 2021 Jun 30;10(7):1644. doi: 10.3390/cells10071644 (PMC8305988; doi:10.3390/cells10071644)
Supplement: Supplementary file 1 [file cells-10-01644-s001.zip › cells-1242539-supplementary.pdf]

SUPPLEMENTARY MATERIAL

(a)

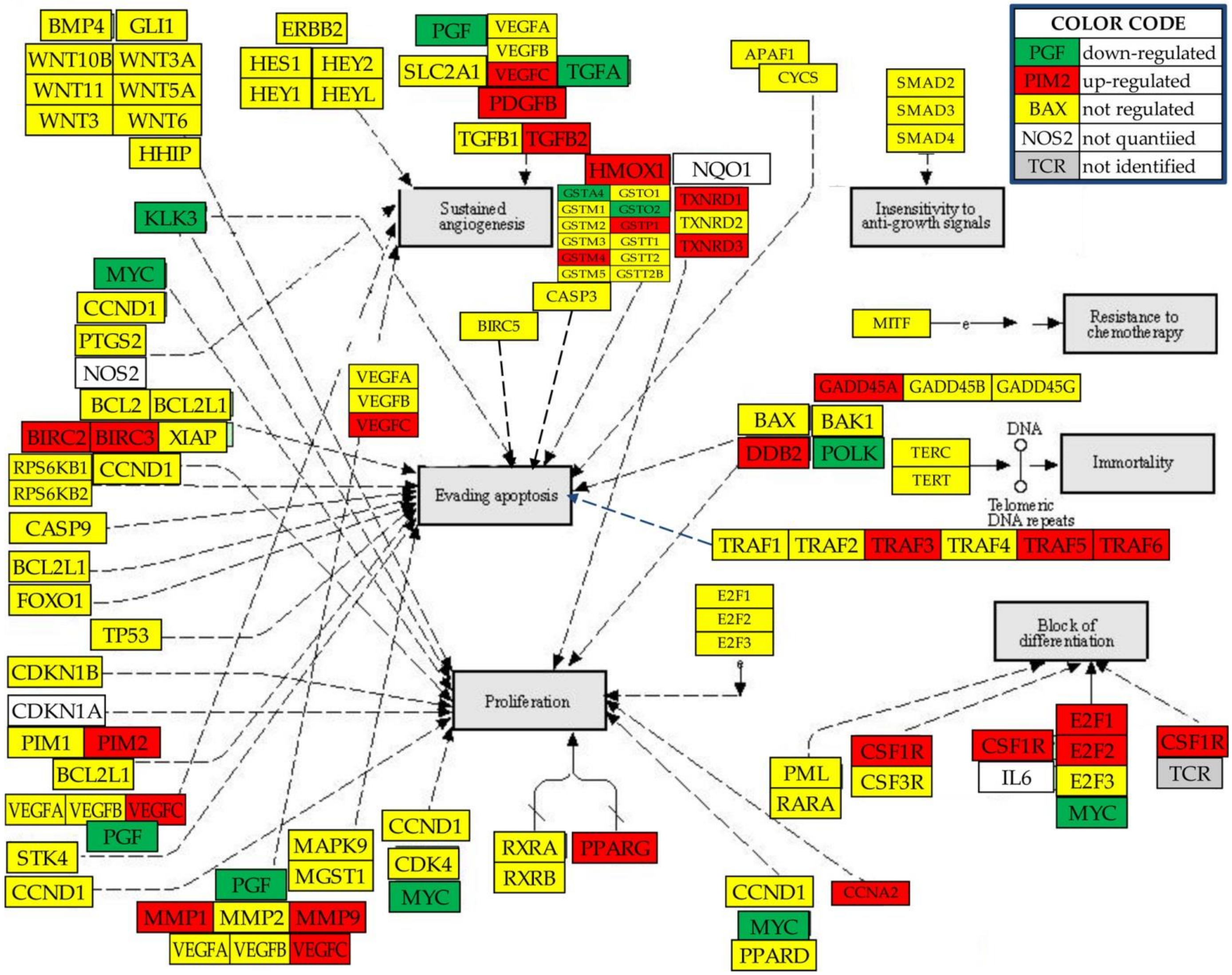

(b)

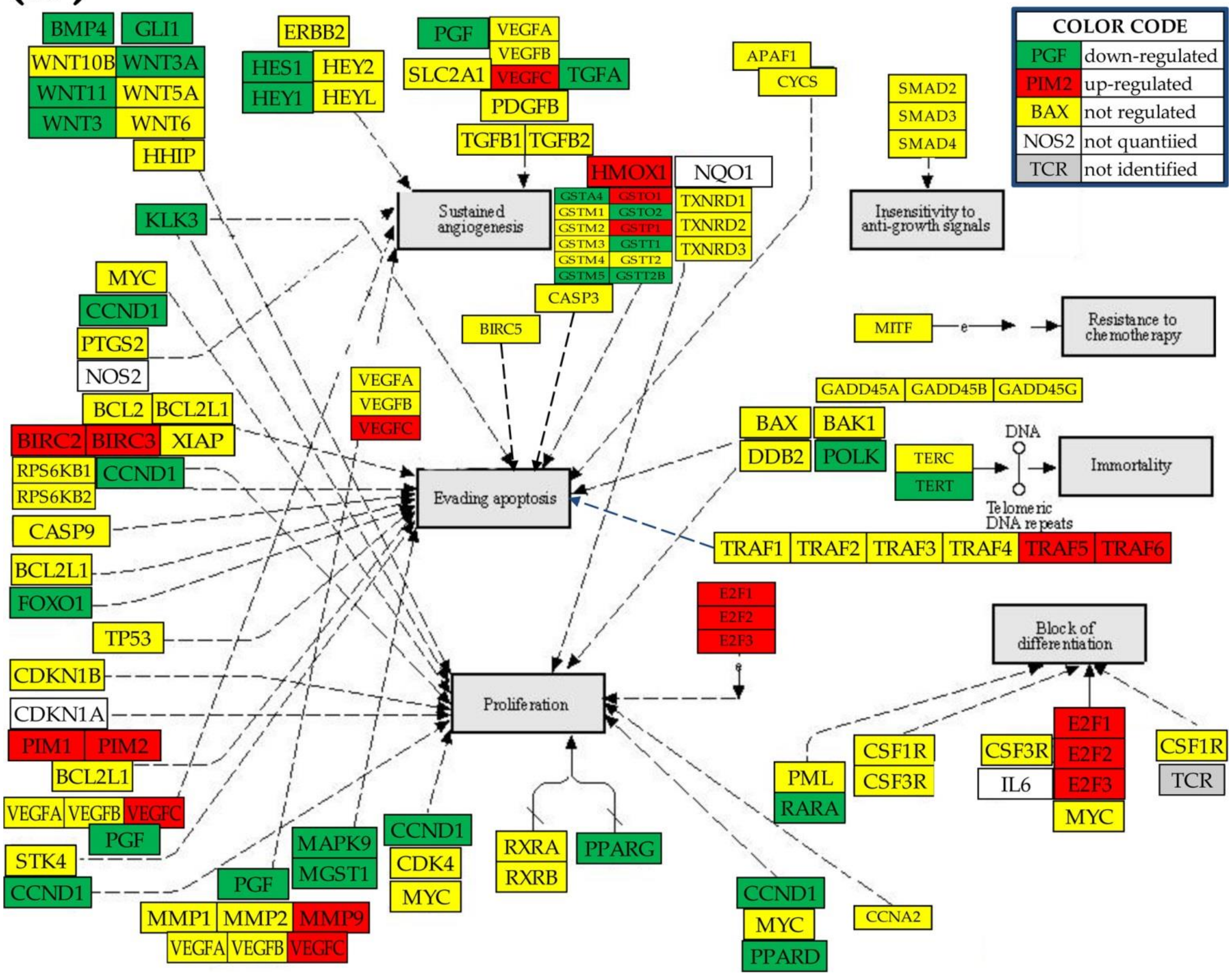
COLOR CODE
PGF down-regulated
PIM2 up-regulated
BAX not regulated
NOS2 not quantiied
TCR not identified
Sustained angiogenesis
Insensitivity to anti-growth signals
Resistance to chemotherapy
Evading apoptosis
Immortality
Proliferation
Block of differentiation
DNA
Telomeric DNA repeats
BMP4
GLI1
WNT10B
WNT3A
WNT11
WNT5A
WNT3
WNT6
HHIP
ERBB2
HES1
HEY2
HEY1
HEYL
PGF
VEGFA
VEGFB
VEGFC
SLC2A1
TGFA
PDGFB
TGFB1
TGFB2
HMOX1
NQO1
GSTA4
GSTO1
GSTM1
GSTO2
GSTM2
GSTP1
GSTM3
GSTT1
GSTM4
GSTT2
GSTM5
GSTT2B
TXNRD1
TXNRD2
TXNRD3
APAF1
CYCS
SMAD2
SMAD3
SMAD4
KLK3
MYC
CCND1
PTGS2
NOS2
CASP3
BIRC5
MITF
BCL2
BCL2L1
BIRC2
BIRC3
XIAP
RPS6KB1
RPS6KB2
CASP9
FOXO1
TP53
CDKN1B
CDKN1A
PIM1
PIM2
STK4
GADD45A
GADD45B
GADD45G
BAX
BAK1
DDB2
POLK
TERC
TERT
TRAF1
TRAF2
TRAF3
TRAF4
TRAF5
TRAF6
E2F1
E2F2
E2F3
MAPK9
MGST1
CDK4
MMP1
MMP2
MMP9
RXRA
RXRB
PPARG
PPARD
CCNA2
PML
RARA
CSF1R
CSF3R
IL6
TCR

(c)

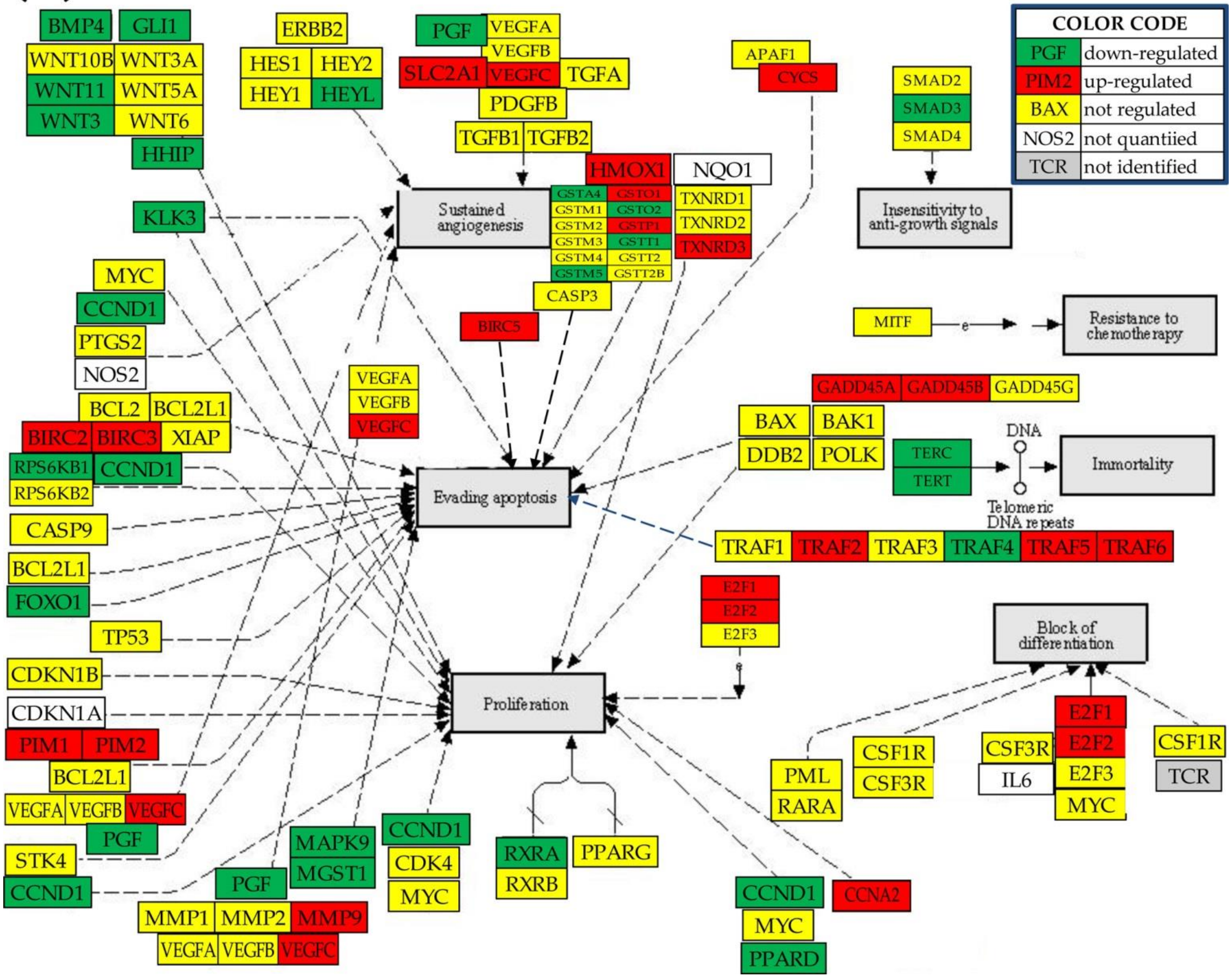

Figure S1. Regulation of genes identified by KEGG as associated with cancer cell survival and proliferation. (**a**) Nodule "A"; (**b**) Nodule "B"; (**c**) Nodule "C". Regulated genes: *BIRC2/3/5* (baculoviral IAP repeat containing 2/3/5), *BMP4* (bone morphogenetic protein 4), *CCNA2/D1* (cyclin A2/D1), *CSFIR* (colony stimulating factor 1 receptor), *DDB2* (damage-specific DNA binding protein 2, 48kDa), *E2F1/2/3* (E2F transcription factor 1/2/3), *FOXO1* (forkhead box O1), *GLI1* (GLI family zinc finger 1), *GSTA4/M1/M4/M5/O1/O2/P1/T1/T2B* (glutathione S-transferase alpha 4/mu 1/mu 4/mu 5/omega 1/omega 2/pi 1/theta1/theta 2B), *HES1* (hes family bHLH transcription factor 1), *HEY1/L* (hes-related family bHLH transcription factor with YRPW motif 1/like), *HHIP* (hedgehog interacting protein), *HMOX1* (heme oxygenase (decycling) 1), *KLK3*, *MAPK9/10* (mitogen-activated protein kinase 9/10), *MGST3* (microsomal glutathione S-transferase 3), *MMP1/9* (mitogen-activated protein kinase 1/9), *MYC* (v-myc avian myelocytomatosis viral oncogene homolog), *PGF* (placental growth factor), *PIM2* (Pim-2 proto-oncogene, serine/threonine kinase), *RARA* (retinoic acid receptor, alpha), *RPS6KB1* (ribosomal protein S6 kinase, 70 kDa, polypeptide 1), *RXRA* (retinoid X receptor, alpha), *SLC2A1* (solute carrier family 2 (facilitated glucose transporter), member 1), *SMAD3* (SMAD family member 3), *TERC* (telomerase RNA component), *TERT* (telomerase reverse transcriptase), *TGFA/B2* (transforming growth factor, alpha/beta2), *TRAF4/5/6* (TNF receptor-associated factor 4/5/6), *TXNRD3* (thioredoxin reductase 3), *VEGFC* (vascular endothelial growth factor C), *WNT3/11/3A* (wingless-type MMTV integration site family, member 3/11/3A).

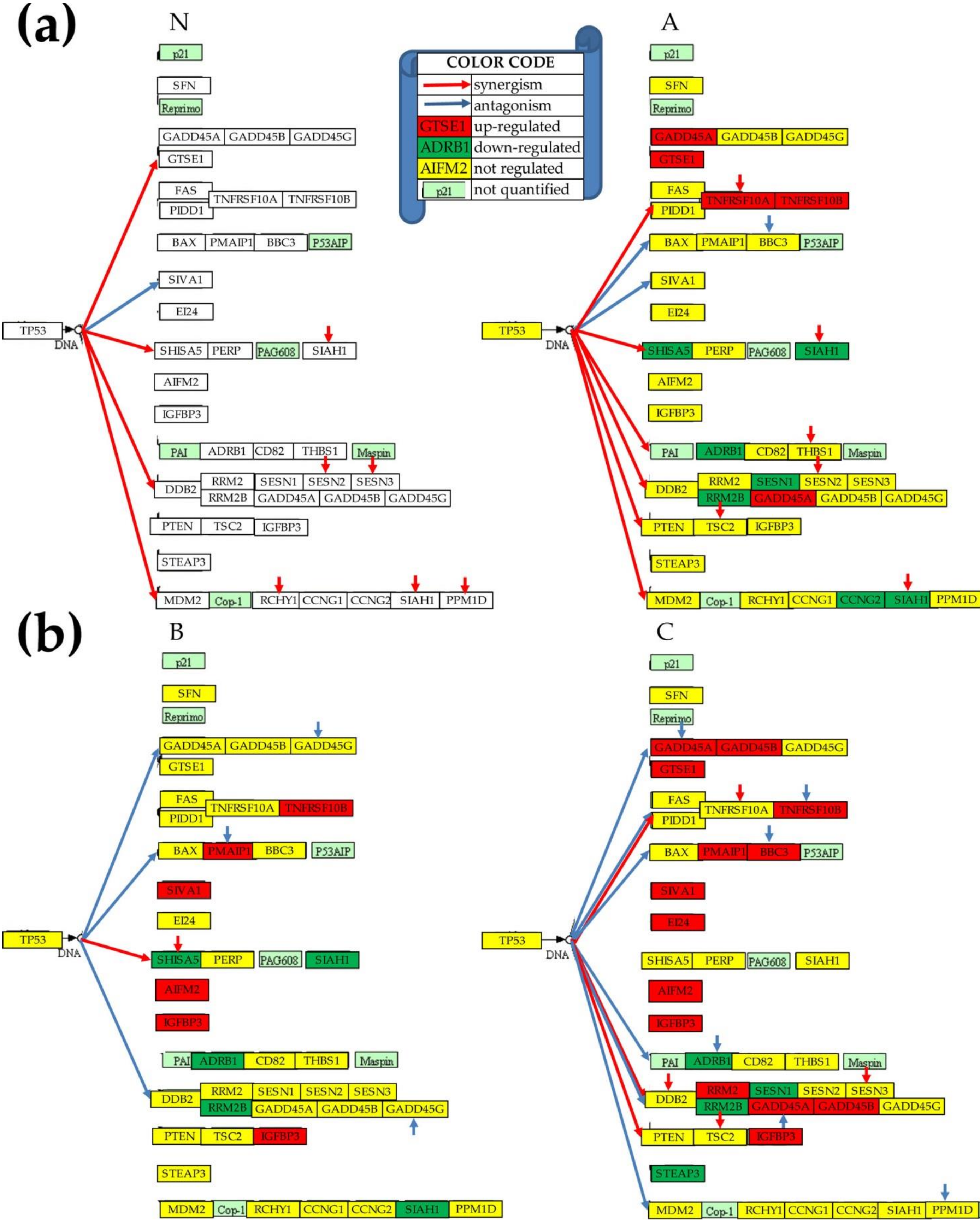

(a)
N
A
COLOR CODE
synergism
antagonism
GTSE1 up-regulated
ADRB1 down-regulated
AIFM2 not regulated
p21 not quantified
TP53
DNA
p21
SFN
Reprimo
GADD45A GADD45B GADD45G
GTSE1
FAS
TNFRSF10A TNFRSF10B
PIDD1
BAX PMAIP1 BBC3 P53AIP
SIVA1
EI24
SHISA5 PERP PAG608 SIAH1
AIFM2
IGFBP3
PAI ADRB1 CD82 THBS1 Maspin
DDB2
RRM2 SESN1 SESN2 SESN3
RRM2B GADD45A GADD45B GADD45G
PTEN TSC2 IGFBP3
STEAP3
MDM2 Cop-1 RCHY1 CCNG1 CCNG2 SIAH1 PPM1D
(b)
B
C

Figure S2. Expression coordination of TP53 with KEGG-determined targeted genes in (**a**) normal tissue; (**b**) cancer nodule "A"; (**c**) cancer nodule "B"; (**d**) cancer nodule "C". Red/blue long arrows indicate the overall synergistic/antagonistic coordination of the block, while short arrows pont to the significantly coordinated genes within the corresponding group of genes. Red/green/yellow background of a gene symbol in a cancer region indicates whether that gene was up-/down- or not significantly regulated in the represented cancer region with respect to the normal tissue. Quantified genes in "N" have the symbols in white background. Light green background of a block indicates that no gene was quantified in that block.

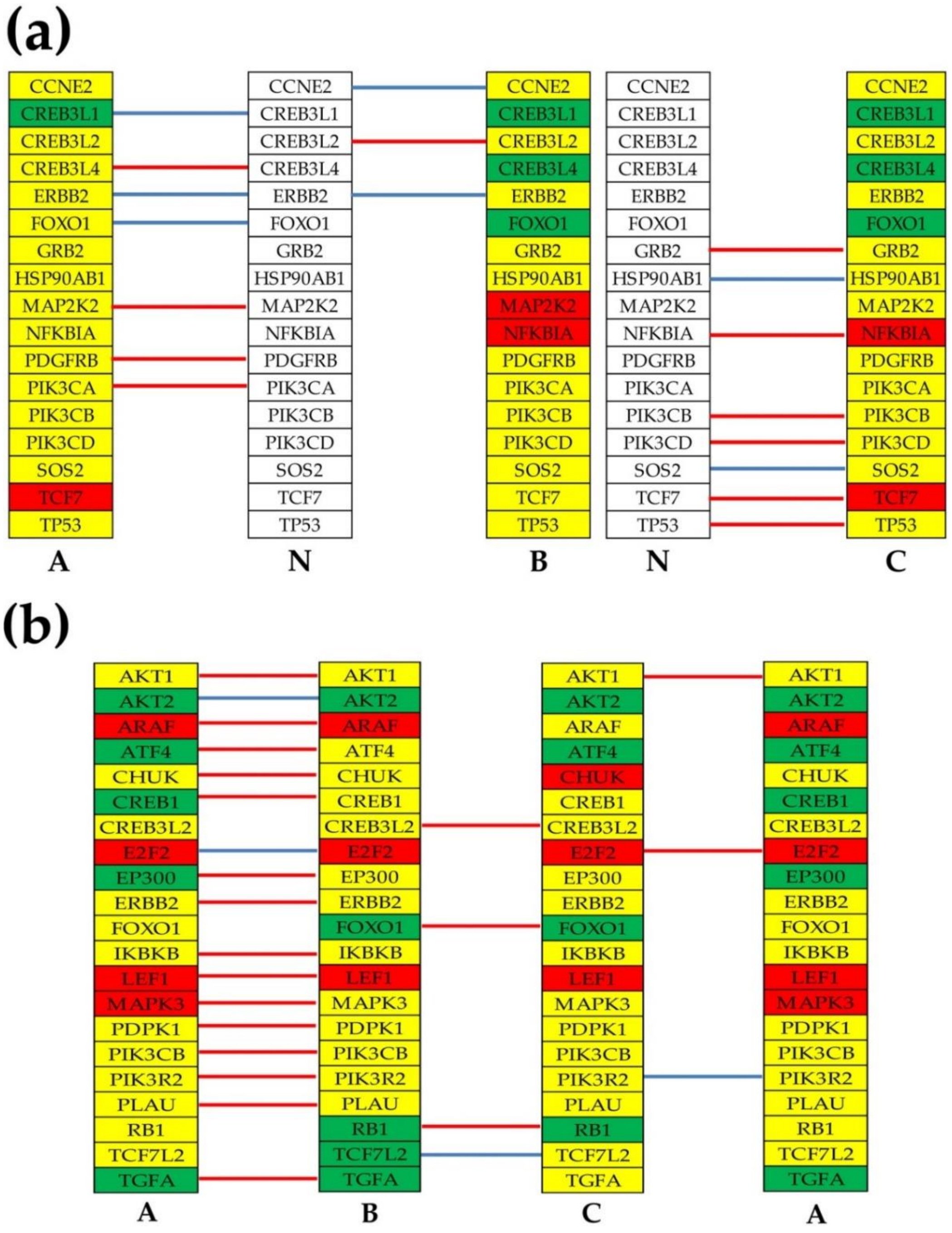

Figure S3. Statistically ($p < 0.05$) significantly in-phase (synchronously) and in-antiphase expressed KEGG-determined prostate cancer genes [37] in pairs of profiled regions. (**a**) Synchrony and anti-synchrony of gene expression profiles in the normal tissue with each cancer nodule. (**b**) Synchrony and-anti synchrony of gene expressions among cancer nodules. Red/blue lines indicate significant synergistic/antagonistic expression of that gene in the paired regions. Red/green/yellow background of a gene symbol in a cancer region indicates whether that gene was up-/down- or not significantly regulated in the represented cancer region with respect to the normal tissue. Quantified genes in "N" have the symbols in white background.
